# Assessment of copy number variation in genes related to drug resistance in *Plasmodium vivax* and *Plasmodium falciparum* isolates from the Brazilian Amazon and a systematic review of the literature

**DOI:** 10.1186/s12936-017-1806-z

**Published:** 2017-04-19

**Authors:** Gabriel Luíz Costa, Lara Cotta Amaral, Cor Jesus Fernandes Fontes, Luzia Helena Carvalho, Cristiana Ferreira Alves de Brito, Taís Nóbrega de Sousa

**Affiliations:** 1 Molecular Biology and Malaria Immunology Research Group, Centro de Pesquisas René Rachou, Fundação Oswaldo Cruz (FIOCRUZ), Belo Horizonte, Minas Gerais Brazil; 2 Hospital Julio Muller, Universidade Federal de Mato Grosso, Cuiabá, Mato Grosso Brazil

**Keywords:** Malaria, *Plasmodium falciparum*, *Plasmodium vivax*, Drug resistance, Copy number variation, Single nucleotide polymorphism, Systematic review

## Abstract

**Background:**

Parasite resistance to anti-malarials represents a great obstacle for malaria elimination. The majority of studies have investigated the association between single-nucleotide polymorphisms (SNPs) and drug resistance; however, it is becoming clear that the copy number variation (CNV) is also associated with this parasite phenotype. To provide a baseline for molecular surveillance of anti-malarial drug resistance in the Brazilian Amazon, the present study characterized the genetic profile of both markers in the most common genes associated with drug resistance in *Plasmodium falciparum* and *Plasmodium vivax* isolates. Additionally, these data were compared to data published elsewhere applying a systematic review of the literature published over a 20-year time period.

**Methods:**

The genomic DNA of 67 patients infected by *P. falciparum* and *P. vivax* from three Brazilian States was obtained between 2002 and 2012. CNV in *P. falciparum* multidrug resistance gene-1 (*pfmdr1*), GTP cyclohydrolase 1 (*pfgch1*) and *P. vivax* multidrug resistance gene-1 (*pvmdr1*) were assessed by real-time PCR assays. SNPs in the *pfmdr1* and *pfcrt* genes were assessed by PCR–RFLP. A literature search for studies that analysed CNP in the same genes of *P. falciparum* and *P. vivax* was conducted between May 2014 and March 2017 across four databases.

**Results:**

All analysed samples of *P. falciparum* carried only one copy of *pfmdr1* or *pfgch1*. Although the *pfcrt* K76T polymorphism, a determinant of CQ resistance, was present in all samples genotyped, the *pfmdr1* N86Y was absent. For *P. vivax* isolates, an amplification rate of 20% was found for the *pvmdr1* gene. The results of the study are in agreement with the low amplification rates for *pfmdr1* gene evidenced in the Americas and Africa, while higher rates have been described in Southeast Asia. For *P. vivax*, very low rates of amplification for *pvmdr1* have been described worldwide, with exceptions in French Guiana, Cambodia, Thailand and Brazil.

**Conclusions:**

The present study was the first to evaluate *gch1* CNV in *P. falciparum* isolates from Brazil, showing an absence of amplification of this gene more than 20 years after the withdrawal of the Brazilian antifolates therapeutic scheme. Furthermore, the rate of *pvmdr1* amplification was significantly higher than that previously reported for isolates circulating in Northern Brazil.

**Electronic supplementary material:**

The online version of this article (doi:10.1186/s12936-017-1806-z) contains supplementary material, which is available to authorized users.

## Background

Malaria is an endemic disease and 3.2 billion people are exposed to its transmission in 95 countries and territories around the world. This disease is caused by five species of *Plasmodium,* with *Plasmodium vivax* having a wider geographic distribution and *Plasmodium falciparum* responsible for the most severe malaria cases associated with high mortality rates [1]. According to the latest estimates, in 2015, approximately 143,000 cases were registered in the Brazilian Amazon region, with 87% of cases due to *P. vivax* [2].

One of the greatest challenges of malaria control is the parasite’s resistance to anti-malarials, defined as the ability of a parasite to survive and/or multiply in the presence of a drug [3]. In Brazil, the first case of drug resistance in *P. falciparum* malaria was reported in the early nineteenth century, to quinine (QN), the first drug used for malaria treatment [4]. It was only in the 1960s that an increased number of cases of *P. falciparum* chloroquine (CQ) resistance began to be reported in the country, concomitant with other countries from South America and Asia [5, 6]. In the 1970s, the Brazilian government adopted sulfadoxine-pyrimethamine (SP) as the first-line treatment; however, in the late 1980s, the rate of SP resistance was close to 90% [7]. From the late 1980s to 2000, Brazil applied various therapeutic schemes, and in 2007 the artemisinin-based combination therapy (ACT) was adopted according to the WHO recommendations to treat uncomplicated *P. falciparum* malaria [6]. Despite the reports of low susceptibility in Asia [8–11] and South America [12], ACT remains effective in Brazil [13]. Concerning *P. vivax* malaria, even though this species has been the most prevalent in Brazil for decades, it is only in the last few years that this species has developed low levels of drug resistance [14–17] and cases of severe malaria [18, 19] have been reported in the Amazon region. Thus, CQ plus primaquine (PQ) is still effective in this area [20].

Anti-malarial resistance studies and surveillance can be conducted using in vitro parasite culture, animal models, in vivo methods and molecular genotyping [21]. The latter aims to identify and monitor genetic polymorphisms related to parasite resistance. Such markers are mainly SNPs, which are characterized by the substitution of a single nucleotide, and CNVs. CNV refers to an increase or decrease in the copy number of a gene in the genome; CNVs are extensively found in humans, mice, *Drosophila* and other eukaryotes [22, 23].

The importance of copy number variation in the *Plasmodium* genome was noticed when an elevated copy number of the *mdr1* (*multidrug resistance gene*-*1*) gene was reported and associated with multidrug resistance in *P. falciparum* isolates in Asia in the late 1980s [24]. The *pfmdr1* gene encodes a protein localized in the parasite digestive vacuole named P-glycoprotein homolog 1 (P-gh1), and many studies have shown a strong association between *pfmdr1* and the multidrug resistance phenotype, such as to mefloquine (MQ), QN and halofantrine (HF) [25–27]. Another gene related to SP resistance in *P. falciparum* and characterized by copy number variation is *gch1* (*GTP cyclohydrolase 1*), a gene encoding the enzyme GTP cyclohydrolase 1 that catalyzes the first step in the folate biosynthesis pathway [28–30]. Regarding CNV in *P. vivax*, the amplification of *pvmdr1*, orthologous to *pfmdr1*, seems to also be related to drug resistance [31, 32]. Due to the challenges of conducting in vitro studies in *P. vivax*, the understanding concerning the resistance to anti-malarials for this species is less comprehensive.

Despite the increased importance of CNV over the last decades, SNPs are the first polymorphisms associated with drug resistance in *Plasmodium*. In *P. falciparum* some SNPs have a major role in the drug resistance phenotype, e.g., K76T polymorphism in the *pfcrt* (*P. falciparum chloroquine*-*resistant transporter*) gene and N86Y, Y184F, S1034C, N1042D, D1246Y in the *pfmdr1* gene [33, 34], while only the mutation Y976F in the *pvmdr1* gene was related to clinical resistance to CQ in *P. vivax* in Southeast Asia and Papua New Guinea [35–37].

Many questions remain about the genetic basis of resistance in *P. falciparum* and *P. vivax*. A majority of studies has investigated the association between SNPs in the genome of *Plasmodium* and resistance to anti-malarials. However, it has become clear that the copy number variation is also associated with this parasite phenotype. The present study characterized the genetic profile of CNV in genes associated with drug resistance in *P. falciparum* and *P. vivax* isolates to provide a baseline for molecular surveillance of anti-malarial drug resistance in the Brazilian Amazon region. Moreover, SNPs in the same *P. falciparum* genes were assayed and correlated to the genetic profile observed for CNV. Finally, the genetic variability described in Brazilian isolates was compared to data published elsewhere applying a systematic review of the literature.

## Methods

### Subjects and sample collection

Sixty-seven patients infected by *P. vivax* or *P. falciparum* from three Brazilian states (31 from Mato Grosso, 26 from Rondônia and 10 from Amapá) were included in this study (Table 1). Epidemiological data indicate that malaria transmission in these areas is generally hypo- to meso-endemic [13]. In Rondônia and Amapá, the individuals were locally infected. However, individuals from Mato Grosso may have acquired the infection in other localities of the Amazon region because there is no transmission in the municipality of blood collection. The eligibility criteria included (i) patients with symptomatic *P. vivax* or *P. falciparum* single infection with any parasitaemia by microscopic examination; (ii) the absence of severe complications of malaria; and, (iii) if the patient was female, the absence of pregnancy. The confirmation of *Plasmodium* spp. infection by microscopy was based on Giemsa-stained thick blood smears evaluated by well-trained microscopists in accordance with the malaria diagnosis guidelines of the Brazilian Ministry of Health. DNA samples were extracted from peripheral blood collected in EDTA-containing tubes for parasite genomic analysis using QIAamp DNA kit (QIAGEN, Chatsworth, CA, USA). Samples were collected between August 2002 and May 2012. Molecular detection and identification of *Plasmodium* species were confirmed later by nested PCR amplification of the 18S rRNA gene in the laboratory to exclude mixed malaria infections [38].

**Table 1:** Description of samples included in this study by period of collection, gender, age and parasitemia

Region (N)^b^	Period	Gender (male, %)	Median age, years (IQR^c^)	Parasitemia, geometric mean (range)^d^
Patients infected by *P. falciparum* ^a^				
Amapá (10)	2004–2005	60	35 (27–46)	1306 (605–2500)
Rondônia (4)	2008	75	42 (32–52)	5004 (1520–24,650)
Mato Grosso (18)	2002–2012	83	36 (26–43)	1505 (62–56,660)
Patients infected by *P. vivax* ^a^				
Mato Grosso (13)	2005–2012	92	35 (17–49)	4816 (992–17,500)
Rondônia (22)	2008	73	41 (23–50)	1840 (95–10,770)

^a^ First line treatment for uncomplicated *P. falciparum* malaria during sample collection: QN + doxycycline + PQ (2001–2007) and ACT (after 2007). Treatment scheme for *P. vivax* therapy: CQ + PQ [6]

^b^ State of sample collection and number of isolates analysed

^c^ Interquartile range

^d^ Parasites/µL

### Copy number estimation of *Plasmodium spp.* genes

The copy number variation of *Plasmodium* genes was determined by quantitative real-time PCR (qPCR) using specific hydrolysis probes and oligonucleotide primers for each gene. Probes and primers previously described were used for amplification of *pfmdr1* [27], *pfgch1* [28] and *pvmdr1* [31]. Amplification reactions were performed in a total volume of 10 µL in the presence of 5 µL of Taqman^*®*^ Universal PCR Master Mix 2× (Applied Biosystems, AB, Foster City, CA, USA), 1 µL DNA (≈100 ng/µL), 900 nM (*pfmdr1*, *pfgch1* and *pvmdr1*) or 300 nM (*pftubulin* and *pvtubulin*) of forward primer, 900 nM of reverse primer (for all genes), and 200 nM (*pfmdr1*, *pfgch1* and *pvmdr1*) or 250 nM (*pftubulin* and *pvtubulin*) of the probe. The cycling parameters for PCR were as follows: initial denaturation at 95 °C for 10 min, 40 cycles of 15 s at 95 °C and 1 min at 60 °C. The PCR was performed in triplicate in the Applied Biosystems Viia7 real-time PCR system (AB) in 384 plates. The single-copy β-tubulin gene was used as a reference gene (normalizer) [27, 31], and a field sample with a single copy of the target gene was used as a calibrator. The ΔΔCt method was used to estimate the copy number of *pfmdr1* and *pvmdr1* genes relative to a standard calibrator sample. For the *pfgch1* gene, a calibration curve was used with plasmids containing the *pfgch1* insert. The samples were considered to have a copy number equal to 1 when the value of the relative quantification was between 0.5 and 1.5, and values with a minimum relative quantification >1.5 were defined as amplified. Only samples with a cycle threshold <32 and a Ct standard deviation <0.3 were considered in the analysis. Each experiment was performed in triplicate, and gene amplification was determined by at least two independent experiments. The accuracy of the qPCR assay was determined from at least three independent experiments, each performed in triplicate, of 19% of the total samples assayed and for the reference laboratory isolate W2mef (2 copies for *pfmdr1* and 1 copy for *pfgch1*) (Additional file 1).

### Detection of single nucleotide polymorphisms in the *pfcrt* and *pfmdr1* genes

PCR followed by restriction fragment length polymorphism analysis (RFLP) was performed to identify SNPs at codons 76 of *pfcrt* and 86 of *pfmdr1*, according to Durand et al. [39] and Lopes et al. [40], respectively. The amplicons were submitted to enzyme digestion with *Apo*I (New England BioLabs, Ipswich, MA, USA) for 2 h at 50 °C. Then, the enzyme was inactivated by exposure to a temperature of 80 °C for 20 min at and the fragments were visualized on a silver-stained 12% polyacrylamide gel.

### Systematic review

A literature search was conducted between May 2014 and March 2017. An advanced search was made using key words (*Plasmodium*, copy number, resistance, gene, copy number resistance and gene copy). These key words were identified in the title, abstract and text, according the search algorithm of each database. Manual exclusion criteria included: studies with other *Plasmodium* species than *P. falciparum* and *P. vivax*; studies with laboratory strains grown in vitro; other systematic reviews; and studies with genes related to anti-malarial resistance other than *pfmdr1*, *pfgch1* and *pvmdr1*. All duplicate articles and review articles were excluded; the clinical studies were included in this systematic review. The title, abstract and methods section of all 309 articles (Additional file 2) were scanned to identify the studies according to the inclusion criteria. From the eligible studies the following data were extracted: author, year of sample collection (when available), country where the study was performed, sample number and the frequency of CNV in parasite population by gene assayed.

## Results and discussion

### Copy number estimation of *mdr1* and *gch1* of *Plasmodium falciparum*

**Fig. 1:**
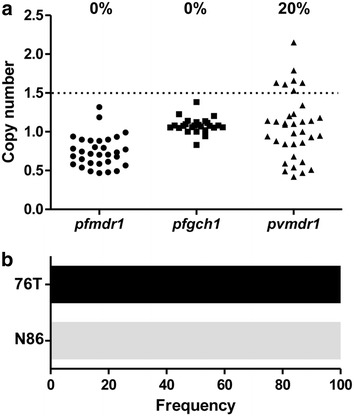
Polymorphism analysis in genes of *P. falciparum* and *P. vivax* isolates from different Brazilian Amazon regions. **a** Copy number variation in genes of *P. falciparum* and *P. vivax*. Only samples with relative quantification above 1.5 were considered amplified. Amplification rates for each gene are indicated in the graph. **b** Frequency of the 76T mutant allele in *pfcrt* and N86 wild-type allele in *pfmdr1*. The frequencies of wild-type and mutated alleles are shown in *black* and *gray*, respectively

31 samples of patients infected by *P. falciparum* were evaluated for copy number variation in the *pfmdr1* gene and 25 samples for *pfgch1*. Parasite isolates were sampled in three regions of Brazil (the Mato Grosso, Rondônia and Amapá states) over a period of 10 years (Table 1). For both genes analysed in this study all *P. falciparum* isolates carried only one copy of the gene (Fig. 1; Additional file 3A, B).

Polymorphisms in *pfmdr1* are related to resistance to several anti-malarials, including CQ, MQ, QN and HF [41]. An increase in the copy number of this gene is the most important determinant of resistance to MQ and to reduced artesunate sensitivity in vitro [26, 27, 42]. In Brazil, *P. falciparum* resistance to anti-malarials has been reported since 1910 (QN—1910; CQ—1960s; SP—1980s; amodiaquine and MQ—1990s) [6]. Another study also assessed the presence of CNV in the *pfmdr1* gene from samples collected over four decades in Brazil (from 1984 to 2011), in which patients were treated with QN plus tetracycline, MQ or ACT [43]. During that period, the rate of *pfmdr1* amplification reached 42%. Particularly, the usage of drugs such as MQ, which is related to selection of resistant parasites with *pfmdr1* amplification, could be an explanation for the observed high rates of amplification. A noticeable difference between the present study and that by Inoue et al. is the region and the period of sample collection (2000s herein), which could lead to the observed differences in genetic diversity. Accordingly, geographical foci of *P. falciparum* with distinct population structures have been described in the Brazilian Amazon region [44]. However, as data from the previous study have not been shown by region, differences in CNV distribution due to the location of the infection could not be assessed here. Alternatively, cases of a decline in resistant isolates by the withdrawal of drug pressure could have occurred as previously reported in Africa [45, 46]. The genome of *P. falciparum* is known to be plastic [47], which means that the parasite can experience rapid and extensive variation in response to changes in its environment; this is also known as phenotypic plasticity [48]. In a short timescale, this phenotypic plasticity can modify or produce new phenotypes. Thus, this phenomenon enables parasites to maximize fitness by mechanisms such as differential gene expression [49]. An example was shown by Preechapornkul et al. where strains containing a single copy of the gene preferentially survived in the absence of MQ compared to those who had multiple copies [25]. Parasites that had fewer copies had a greater survival rate due to this energy demand generated by P-gh1. This protein acts as an efflux pump that transports substrates against a concentration gradient with ATP hydrolysis [50]. In Brazil, MQ was officially introduced in 1987 for the treatment of CQ- and SP-resistant *P. falciparum*, and few years later, in 2001, MQ was substituted with the combination of QN plus doxycycline, followed by primaquine (PQ) as a first line therapy [6]. At this time, MQ plus PQ was instituted as a second line drug combination, and only after 2006 did the ACT become adopted in Brazil [6]. Thus, the lower pressure exerted by MQ may have led to selection of parasites bearing no genetic amplification after the first decade of the 2000s.

*Plasmodium falciparum* resistance to SP is associated with SNPs in two genes in the folate pathway, *dihydrofolate reductase* (*dhfr*) and *dihydropteroate synthetase* (*dhps*) [51, 52]. However, polymorphisms in these genes may alter their efficiencies. The *pfgch1* gene encodes the first enzyme of this pathway and an increased copy number of this gene may act as a compensatory mechanism since it is associated with a greater number of point mutations in *dhfr* and *dhps* [28, 53, 54]. Few studies have evaluated the role of CNV in resistance to SP, particularly in the Americas. The present study was the first to evaluate *gch1* CNV in *P. falciparum* isolates from Brazil. Samples analysed here date from the 2000s, more than twenty years after the withdrawal of SP in the Brazilian therapeutic scheme in the late 1980s. Thus, the absence of amplification of the *pfgch1* gene in the Brazilian isolates may be the result of antifolates withdrawal, which could have favored the spread of parasites bearing a single copy of the gene. In fact, the presence of CNV in this gene seems to not be advantageous in the absence of the drug pressure, having impact on parasite growth [53]. Still, since the evolution of antifolate-resistant parasites is multifaceted and complex and the activity of the *gch1* gene is linked with other enzymes (e.g., *dhps* and *dhfr*), further analysis is required to confirm the significance of *gch1* CNV on the gain in SP resistance.

### Analysis of single nucleotide polymorphisms in the *crt* and *mdr1* genes of *Plasmodium falciparum*

The samples of *P. falciparum* were assayed for two polymorphisms in *pfcrt* (K76T) and *pfmdr1* genes (N86Y). Twenty-seven samples (84% [27/32]) were successfully genotyped (10 from Amapá, 16 from Mato Grosso and 1 from Rondônia) for both genes. For *pfcrt* gene, all samples carried the polymorphism at codon 76, while the substitution N86Y in the *pfmdr1* gene was absent (Fig. 1). The fixation of the 76T allele is in agreement with other studies in Brazil [43, 55, 56], despite the withdrawal of CQ from national treatment guidelines in the mid-1980s [57]. The presence of the 76Y allele is in accordance with the description of isolates resistant to CQ in the same regions analysed here [43, 55]. The K76T mutation has been proven essential to CQ resistance, as demonstrated by transfection experiments and it was suggested that this mutation in the *pfcrt* gene confers resistance to CQ by reducing the amount of drug in the digestive vacuole of the parasite [33, 34, 58, 59].

Field studies have observed a significant association between the *pfcrt* (76T) and *pfmdr1* (86T) alleles, suggesting a joint contribution of these two genes to CQ resistance [60, 61]. Reinforcing these findings, the study by Veiga et al. using zinc-finger nucleases to genetically modify the *pfmdr1* gene confirmed the contribution of the N86Y substitution to CQ and amodiaquine resistance. In contrast, this substitution increased parasite susceptibility to MQ and lumefantrine [62]. Additionally, an association between the 86Y mutant and reduced susceptibility to artemether was found using in vitro tests [63], although other studies have reported contradictory results with the N86Y substitution associated with increased artemether sensitivity [45, 64, 65]. In samples analysed here, the N86Y substitution was absent, a similar result to that observed by other studies that have also reported low frequency or absence of this substitution in *pfmdr1* in Brazil [43, 55, 56]. On the other hand, Inoue et al. reported the emergence of the 86Y mutant in 25 and 43% of isolates from Brazil and Guyana, respectively, over the last few decades. These findings led the authors to hypothesize that the increase in prevalence of the 86Y mutant could be related to the indiscriminate use of ACT in Guyana and the high flow of gold mine workers between Brazil and this neighboring country. Although not analysed here, other polymorphisms (e.g. Y184F, S1034C, N1042D and D1246Y) that influence parasite response to different drugs such as CQ, MQ, QN, HF and artemisinin [41, 66, 67] are found with high frequency in South America [43, 55, 68–70]. In this way, it seems that the markers of CQ resistance may differ between South America and other endemic areas. Thus, it is necessary to define better and more reliable polymorphisms to characterize South American samples.

### Copy number estimation of *mdr1* of *Plasmodium vivax*

To characterize the pattern of copy number variation of *P. vivax* isolates from the Brazilian Amazon region, thirty-five samples collected in various time periods and from two different areas (the states of Mato Grosso and Rondônia) were analysed. Overall, an amplification rate of 20% (7/35) was found for the *pvmdr1* gene (Fig. 1). In Mato Grosso, six of 13 (46%) isolates had multiple copies of the *pvmdr1* gene (Additional file 3C). Despite being collected in the state of Mato Grosso, the probable locality of infection for these individuals carrying isolates with multiple copies of *pvmdr1* included others areas of the Amazon region, including the states of Pará and Rondônia, as well as the neighboring country French Guiana. In addition, the majority of these isolates were collected in the 2010s decade. For 22 samples collected in Rondônia only one (4%) had multiple copies of the *pvmdr1* gene (Additional file 3C). Overall, *pvmdr1* copy number variation was not related to parasitaemia (median: 3150 [IQR 2935-12,000 parasites/μ] for individuals carrying isolates with multiple copies vs. 2483 [992-6175 parasites/μ] for samples with one copy of *pvmdr1*; Mann–Whitney U test, *P* = 0.201) or age (mean: 32 [SD 14.8 years] for samples with multiple copies vs. 37 [15.1 years] for samples with one copy of *pvmdr1*; Student’s t test, *P* = 0.469). Among samples with multiple copies of *pvmdr1*, the recurrence of the disease was reported in two individuals from Mato Grosso in a period of 45 days to 3 months after the initial infection.

In Brazil and in several other malaria endemic areas CQ and PQ are still the main drugs used to treat *P. vivax* [71]. However, resistance to CQ has been reported in the Brazilian Amazon [14–17]. Similar to *P. falciparum*, the copy number variation on *pvmdr1* is supposed to be related to drug resistance [31]. In Brazil, only one study described *pvmdr1* copy number variation, showing an amplification rate of 0.9% in isolates from the state of Acre [72]. Furthermore, the authors have not observed any relation between SNPs described in *pvmdr1* and CQ resistance. In this study, *pvmdr1* amplification was observed in 20% of patients infected in different areas of Northern Brazil. One individual was declared to have been infected in French Guiana, where a high number of isolates with multiple copies of the *pvmdr1* gene has previously been reported [73, 74]. The clinical features of age and parasitaemia, which are important predictors of patient’s response to the treatment [27, 75, 76] did not differ between individuals carrying parasites with one or multiple copies of *pvmdr1* in the present study. Thus, for the two individuals bearing isolates with multiple copies of the gene who also had episodes of recurrence, there is the possibility of treatment failure due to parasite resistance to anti-malarials. However, as the individuals were not followed up after drug therapy with plasma drug level and parasitaemia measurements, the authors could not exclude the possibility of inadequate drug absorption. Additionally, the authors could not exclude the possibility of recurrence due to CQ or PQ-impaired metabolism by variants of cytochrome P450 isoenzymes [77], as the genes that encode the enzymes were not assayed here. On the other hand, a new infection seems to be unlikely since these individuals reside in an area without active transmission of malaria and did not travel to other endemic areas in the Brazilian Amazon after their initial infections.

As previously suggested for other geographical locations, *pvmdr1* amplification could be associated with MQ pressure [31, 78] in a mixed infection context, when this drug was used alone or in combination to treat *P. falciparum* malaria. Accordingly, *P. vivax* resistance to MQ has been reported in the Brazilian Amazon region [16, 55, 79]. In *P. falciparum*, resistance to MQ has been associated with increased copy number of *pfmdr1* [27]. The mechanisms of MQ resistance seem to be similar between *P. vivax* and *P. falciparum*. In areas of Southeast Asia with intense and sustained MQ pressure, gene amplifications of *pvmdr1* were significantly more common than in those patients from other localities where there had been less parasite exposure to MQ [31]. Additionally, Suwanarusk et al. reported amplification of 21% in the *pvmdr1* gene in *P. vivax* isolates from Thailand, which was associated with a twofold increase in MQ IC_50_ [32]. Mefloquine and CQ seem to exert selection pressure in opposite direction on *pvmdr1*, with gene amplification associated with an increase of susceptibility to CQ [32]. Therefore, the observed pattern of *pvmdr1* copy number variation in the Brazilian Amazon region and elsewhere would be the result of pressure exerted by the two drugs. For Brazilian samples, an increase in the prevalence of *pvmdr1* amplification was observed over the 7-years period analysed. However, this finding requires further investigation as it might indicate that the *P. vivax* population is undergoing an increased susceptibility to CQ.

### Copy number variation in *Plasmodium* spp.: a systematic review of the literature

A total of 309 articles were selected from four databases (PubMed, Malaria Journal, Science Direct and CAPES), and 82 of these were included in the systematic review according to the established criteria. The data of articles included in this study are available in supplementary material (Additional file 4). Studies from four continents constituted the review, where Asia and Africa were the regions with the majority of selected articles (88%). The majority of the studies were about *P. falciparum*, related to copy number variation in *pfmdr1*. Only two studies analysed the *pfgch1* gene. For the *pvmdr1* gene, eight studies were eligible for inclusion.

**Fig. 2:**
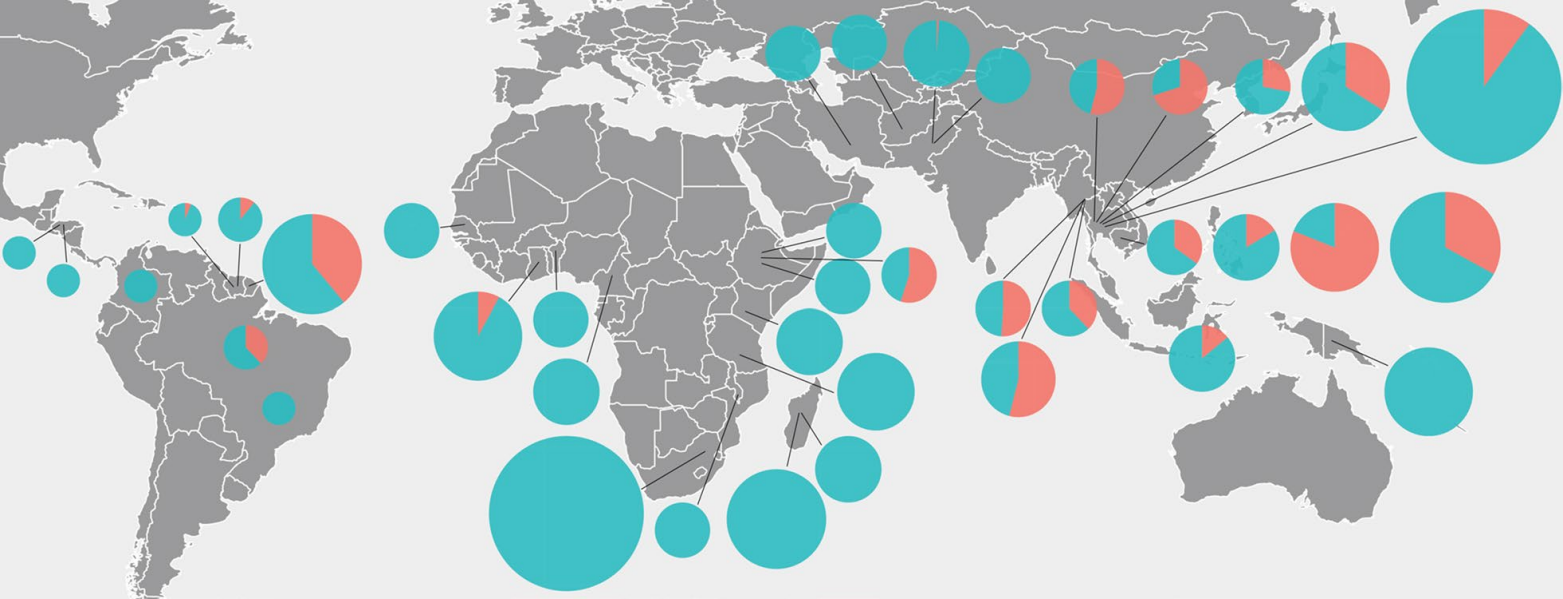
Global pattern of *mdr1* copy number variation in *P. falciparum* isolates. The frequency of isolates carrying one or multiple copies of *pfmdr1* is indicated by *green* and *red* pie charts, respectively. Only the studies that analysed more than 100 isolates are shown in the map. The size of the pie charts is proportional to the number of isolates analysed in each study

The distribution of resistant isolates to anti-malarials is different between the endemic regions of the world, as well as the profile of CNV in genes of the parasite. In the Americas and Africa, the amplification of *pfmdr1* is not common and is restricted to certain localities. Thus, the majority of the studies in the Americas reported an absence of *pfmdr1* amplification in *P. falciparum* isolates; the amplification rates varied from 6 to 38% (Fig. 2; Additional file 4) [43, 69, 80–83]. For some countries such as Brazil and French Guiana, there was approximately 30% *pfmdr1* amplification (between 2 and 4 copies) in the samples analysed. In contrast, in Africa, the prevalence of isolates carrying one copy of *pfmdr1* reached almost 100% in different countries, with the exception of Ethiopia (Fig. 2). Unlike these regions, Asian countries present a high rate and distribution of isolates with *pfmdr1* amplification (Fig. 2). The studies show that on the Thailand-Myanmar border, the amplification rate is greater, reaching 100% [84, 85]. In general, Southeast Asian countries had a greater prevalence of *P. falciparum* isolates with *pfmdr1* amplification, while isolates from Southwest Asia showed no gene amplification [86–90]. Taken together, these differences in gene amplification rates may be the result of the adoption of different treatment regimens for malaria by different countries over the years. In fact, there is evidence that amplification of the *pfmdr1* gene in *P. falciparum* has arisen as multiple independent events, suggesting that this region of the genome is under strong selective pressure [91]. Since gene amplification has a cost to the parasite’s fitness [25, 92], the drug pressure withdrawal could also have favored parasites without gene amplification contributing to the genetic variability observed.

Regarding *pfgch1*, only two studies evaluated the profile of CNV in field isolates. In Africa, Kiwuwa et al. have not detected any amplification in the 21 isolates analysed [93]. However, in Southeast Asia a significant difference in the rates of *pfgch1* amplification was reported for isolates from Thailand (72%) and Laos (2%), countries that have contrasting selection histories with antifolate drugs [28]. Whereas SP was not extensively used in Laos until 2006, despite their use as official second line treatment for malaria [94], Thailand has had a longstanding history of antifolate use [95]. This geographical differentiation on *pfgch1* CNV suggests local adaptations to drug pressure, which has been experimentally corroborated [53].

**Fig. 3:**
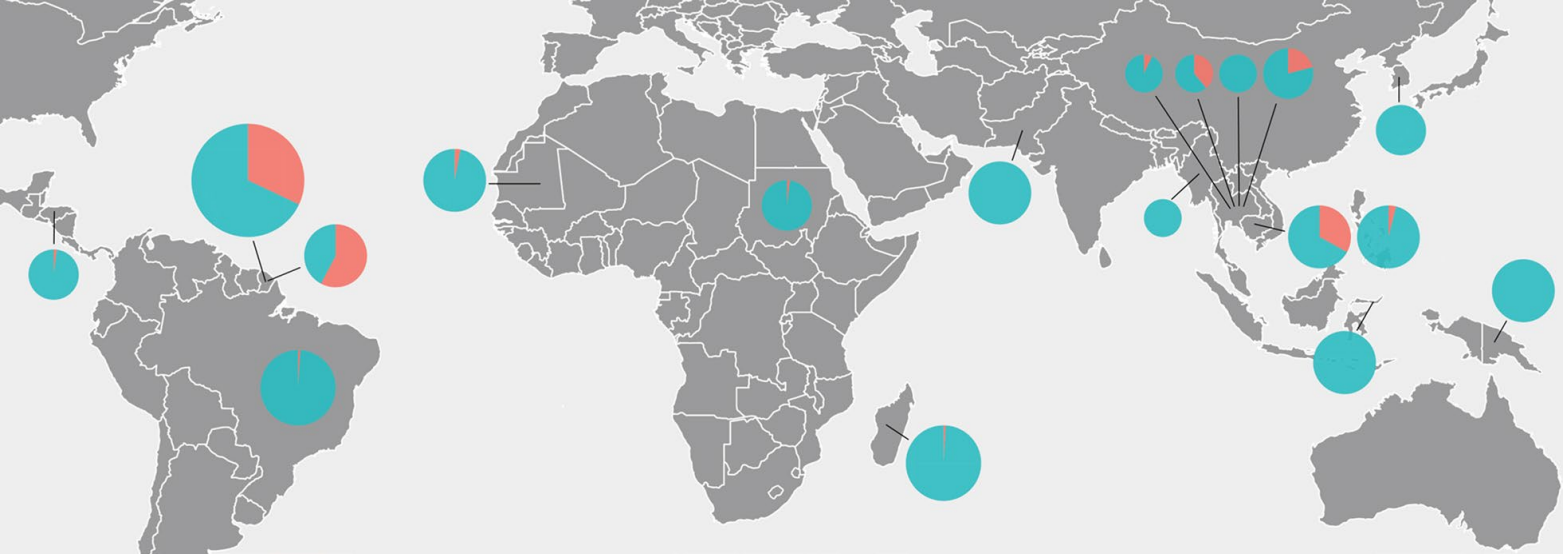
Global pattern of *mdr1* copy number variation in *P. vivax* isolates. The frequency of isolates carrying one or multiple copies of *pvmdr1* is indicated by *green* and *red* pie charts, respectively. The size of the pie charts is proportional to the number of isolates analysed in each study

As described for *P. falciparum*, there is a difference regarding the distribution of CNV allelic frequencies of *P. vivax* isolates in the endemic areas. In the Americas and Africa, only a few studies have assessed *pvmdr1* CNV, finding very low rates of amplification (1–3%) (Fig. 3) [72, 74, 83, 96]. Conversely, a higher rate (up to 59%) of isolates harboring multiple copies of *pvmdr1* was reported in French Guiana, where MQ was widely used alone or in combination with artesunate for treatment of uncomplicated *P. falciparum* malaria, and therefore *P. vivax* were subjected to indirect selection pressure by the drug [73, 74]. In Asia, *pvmdr1* amplification was reported in Cambodia (4 to 33%) [37, 74] and in Thailand (7–39%) [37, 97]. Commonly, in these areas where *pvmdr1* amplification is frequent, there has been intense current or past use of mefloquine to treat uncomplicated *P. falciparum* malaria [37, 74].

## Conclusion

For *P. falciparum* isolates, no amplification was found for *pfmdr1* or *pfgch1*, but the SNP K76T associated with chloroquine resistance was present. Notably, the rate of *pvmdr1* amplification observed in this study was significantly higher than previously reported for isolates circulating in Northern Brazil. Furthermore, a wide variation in the amplification rate of *pvmdr1* was observed between the two study sites in Brazil. In a global view, in the Americas and Africa the amplification rates of the *mdr1* gene of *P. falciparum* were generally very low, with a few exceptions. In Asia, particularly in Thailand and Cambodia, the highest rates of *pfmdr1* amplification were reported. In general, *P. falciparum* showed the highest rates of gene amplification; however, it is important to highlight that especially for *P. vivax*, the available information is restricted and may not reflect the actual picture of genetic variability for this species.

## Additional files

**Additional file 1.** Accuracy of quantitative Real-Time PCR assay to estimate the copy number variation in genes of *Plasmodium falciparum* and *Plasmodium vivax.*

**Additional file 2.** Article selection by preestablished criteria.

**Additional file 3.** Real-time PCR estimates of the relative copy number of *P. falciparum* (A and B) and *P. vivax* genes (C). Collection site of isolates is indicated on the x-axis. Only samples with a minimum relative quantification above 1.5 were considered amplified. RO, Rondônia.

**Additional file 4.** Systematic review result for copy number variation in genes related to drug resistance in *Plasmodium vivax* and *Plasmodium falciparum* isolates worldwide.

## Abbreviations

SNP: single nucleotide polymorphism
CNV: copy number variation
mdr: multidrug resistance gene-1
P-gh1: P-glycoprotein homolog 1
gch1: GTP cyclohydrolase 1
crt: chloroquine-resistant transporter
dhfr: dihydrofolate reductase
dhps: dihydropteroate synthetase
PCR-RFLP: polymerase chain reaction followed by restriction fragment length polymorphism
qPCR: quantitative polymerase chain reaction
CQ: chloroquine
QN: quinine
SP: sulphadoxine–pyrimethamine
ACT: artemisin-based combination therapy
MQ: mefloquine
PQ: primaquine
HF: halofantrine
ATP: adenosine triphosphate
IC_50_: half maximal inhibitory concentration
