# Supplementary material for: Assessment of copy number variation in genes related to drug resistance in Plasmodium vivax and Plasmodium falciparum isolates from the Brazilian Amazon and a systematic review of the literature
Source: Malar J. 2017 Apr 19;16:152. doi: 10.1186/s12936-017-1806-z (PMC5395969; doi:10.1186/s12936-017-1806-z)
Supplement: Supplementary file 1 — Additional file 1. Accuracy of quantitative Real-Time PCR assay to estimate the copy number variation in genes of Plasmodium falciparum and Plasmodium vivax. [file 12936_2017_1806_MOESM1_ESM.pdf]

Additional file 1. Accuracy of quantitative Real-Time PCR assay to estimate the copy number variation in genes of *Plasmodium falciparum* and *Plasmodium vivax*.

Sample ID	Target Name	RQ	RQ Min [1]	RQ Max [1]	RQ Mean [2]	RQ SD [2]	CV (%)
		1.18	0.88	1.59			
1	*pfmdr1*	0.88	0.80	1.04	1.13	0.230	20.3
		1.34	1.08	1.64			
		1.00	0.65	1.54			
2	*pfmdr1*	0.86	0.44	1.66	0.92	0.074	8.1
		0.90	0.60	1.40			
		1.10	0.70	1.60			
3	*pfmdr1*	0.74	0.49	1.13	1.01	0.235	23.3
		1.18	0.97	1.44			
		1.07	0.79	1.45			
4	*pfmdr1*	0.74	0.57	0.94	0.80	0.242	30.0
		0.60	0.42	0.87			
		1.07	0.57	2.00			
5	*pfmdr1*	1.12	0.88	1.42	1.04	0.091	8.7
		0.94	0.66	1.35			
		0.90	0.72	1.12			
6	*pfmdr1*	0.72	0.59	0.88	0.77	0.115	15.0
		0.69	0.44	1.06			
		2.14	1.77	2.59			
7	*pvmdr1*	1.69	1.32	2.16	1.96	0.242	12.3
		2.06	1.73	2.44			
		2.70	2.40	3.10			
8	*pvmdr1*	2.10	1.60	2.90	2.56	0.404	15.8
		2.87	2.47	3.33			
		1.01	0.72	1.42			
		1.96	1.32	2.92			
9	*pvmdr1*	1.75	1.30	2.36	1.44	0.483	33.4
		1.05	0.45	2.45			

10	*pvmdr1*	1.56	1.27	1.92	1.60	0.213	13.3
		1.41	1.11	1.79			
		1.83	1.65	2.03			
11 (W2mef)	*pfmdr1*	3.14	2.20	4.48	2.77	0.427	15.4
		2.87	2.22	3.71			
		2.30	1.90	2.60			

**Sample ID**	**Target Name**	**RQ**	**SD**		**RQ Mean** [2]	**RQ SD** [2]	**CV (%)**
12	*pfgch1*	1.35	0.058		1.22	0.204	16.7
		1.33	0.001				
		0.99	0.017				
13	*pfgch1*	1.35	0.016		1.38	0.386	27.9
		1.79	0.028				
		1.02	0.011				
14	*pfgch1*	1.30	0.010		1.09	0.126	11.6
		1.05	0.019				
		1.07	0.006				
		1.05	0.008				
		0.97	0.014				
15 (W2mef)	*pfgch1*	0.96	0.012		1.02	0.046	4.5
		1.05	0.01				
		1.03	0.01				

RQ, relative quantification; Min, minimum; Max, maximum; SD, standard deviation; CV, coefficient of variation

[1] Each experiment was performed in triplicate and the fold-difference in gene copy number of the sample relative to the calibrator (sample with one copy of the gene) was expressed as a range of relative quantification (RQ min and RQ max).

[2] Mean and standard deviation of the gene copy number estimates (RQ) considering all experiments performed to each sample.
