# Supplementary material for: Assessment of copy number variation in genes related to drug resistance in Plasmodium vivax and Plasmodium falciparum isolates from the Brazilian Amazon and a systematic review of the literature
Source: Malar J. 2017 Apr 19;16:152. doi: 10.1186/s12936-017-1806-z (PMC5395969; doi:10.1186/s12936-017-1806-z)
Supplement: Supplementary file 2 — Additional file 2. Article selection by preestablished criteria. [file 12936_2017_1806_MOESM2_ESM.pdf]

Additional file 2. Article selection by preestablished criteria.

Database	Selected	Excluded	Included
Pubmed[a]	96	41	55
Malaria Journal[b]	135	117	18
Science Direct[c]	27	23	4
CAPES[d]	51	46	5
TOTAL	309	227	82

[a] https://www.ncbi.nlm.nih.gov/pubmed/

[b] https://malariajournal.biomedcentral.com/

[c] http://www.sciencedirect.com/

[d] http://www.capes.gov.br/
