# Supplementary figures and images for: Assessment of copy number variation in genes related to drug resistance in Plasmodium vivax and Plasmodium falciparum isolates from the Brazilian Amazon and a systematic review of the literature
Source: Malar J. 2017 Apr 19;16:152. doi: 10.1186/s12936-017-1806-z (PMC5395969; doi:10.1186/s12936-017-1806-z)

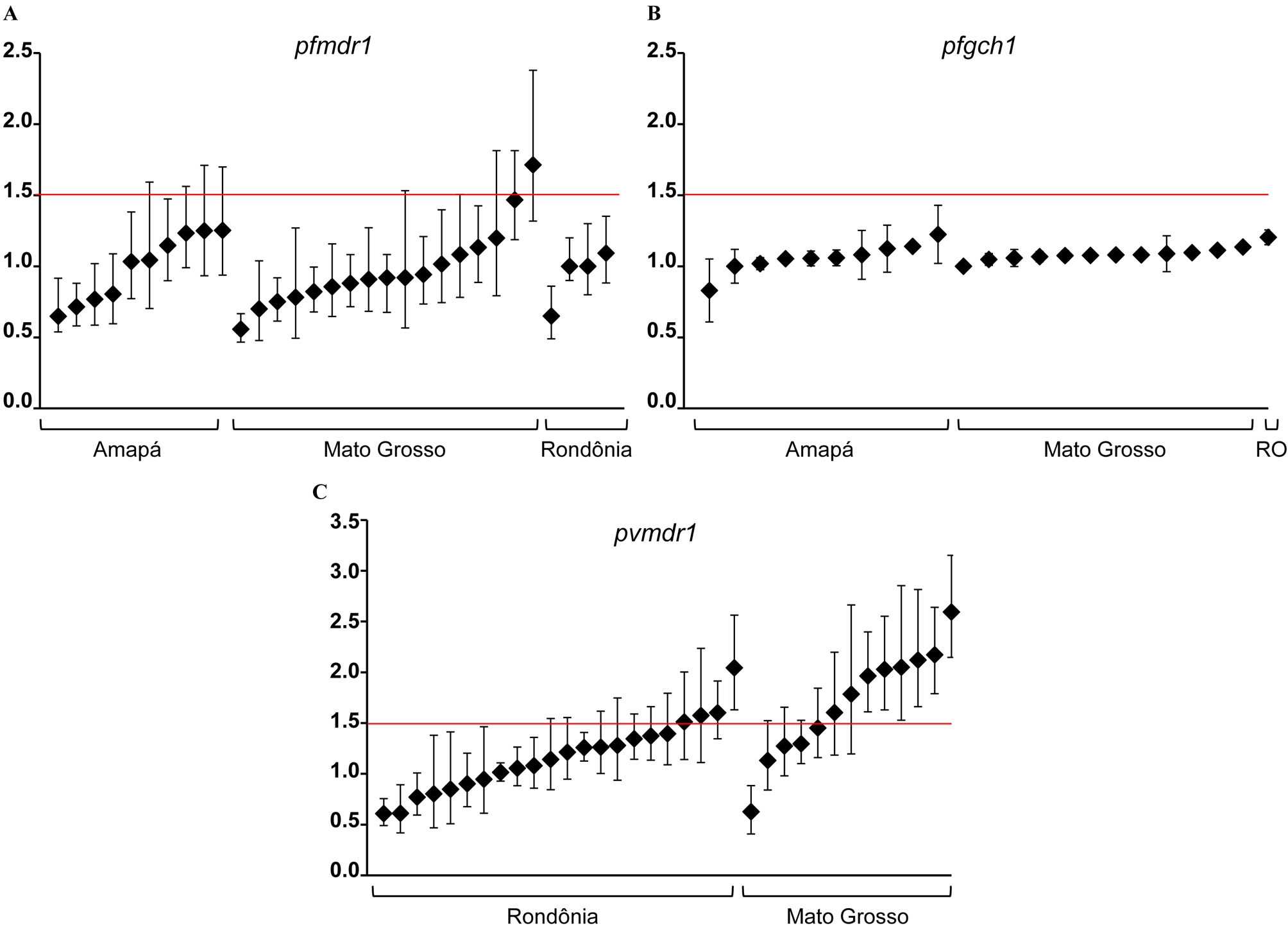

Supplement: Supplementary file 3 — Additional file 3. Real-time PCR estimates of the relative copy number of P. falciparum (A and B) and P. vivax genes (C). Collection site of isolates is indicated on the x-axis. Only samples with a minimum relative quantification above 1.5 were considered amplified. RO, Rondônia. [file 12936_2017_1806_MOESM3_ESM.tif]
