# Supplementary material for: Assessment of copy number variation in genes related to drug resistance in Plasmodium vivax and Plasmodium falciparum isolates from the Brazilian Amazon and a systematic review of the literature
Source: Malar J. 2017 Apr 19;16:152. doi: 10.1186/s12936-017-1806-z (PMC5395969; doi:10.1186/s12936-017-1806-z)
Supplement: Supplementary file 4 — Additional file 4. Systematic review result for copy number variation in genes related to drug resistance in Plasmodium vivax and Plasmodium falciparum isolates worldwide. [file 12936_2017_1806_MOESM4_ESM.pdf]

Additional file 4. Systematic review result for copy number variation (CNV) in genes related to drug resistance in *Plasmodium vivax* and *Plasmodium falciparum* isolates worldwide.

CNV in *pvmdr1*						
**Reference**	**Year**	**Country**	**N** [3]	**Gene Copy Number**		
				**1**	**2 to 4**	**> 4**
		**Americas**				
[72]	2004-2011 [1]	Brazil	215	99%	1%	
[73]	1997-2013 [1]	French Guiana	591	69%	31%	
[74]	2000-2003 [1]	French Guiana	117	41%	56%	3%
[83]	2004-2009 [1]	Honduras	61	98%	2%	
		**Africa**				
[98]	2007-2009 [1]	Mauritania	105	97%	3%	
[74]	2007 [1]	Sudan	59	98%	2%	
	2006-2007 [1]	Madagascar	199	99%	1%	
		**Asia**				
[86]	2011 [1]	Pakistan	108	100%		
[74]	2010 [1]	Cambodia	117	67%	33%	
[97]	2006-2007 [1]	Thailand	27	93%	7%	
	2007-2009 [1]	Republic of Korea	96	100%		
	1999 [1]	Myanmar	11	100%		
[37]	2008 [1]	Cambodia	109	96%	4%	
	2009 [1]	Thailand	49	61%	39%	
[99]	2008 [1]	Thailand	1	100%		
[32]	2003-2007 [1]	Thailand	71	79%	21%	
		Indonesia	114	100%		
		**Oceania**				
[97]	2008 [1]	Papua New Guinea	1	100%		

			**CNV in *pfmdr1***			
			**Americas**			
[80]	1998 [2]	Brazil	11	100%		
[43]	1984-2011 [1]		96	62%	38%	
[81]	2000-2008 [1]	French Guiana	547	61%	35%	4%
[83]	2004-2009 [1]	Honduras	32	100%		
[90]	2004-2009 [1]	Honduras	28	100%		
	1999-2001 [1]	Colombia	44	100%		
[69]	2005-2006 [1]	Suriname	35	94%	6%	
[82]	2005-2009 [1]		63	89%	11%	
			**Africa**			
[90]	1978-1981 [1]	Liberia	30	100%		
	2001-2004 [1]	Guinea Bissau	50	100%		
	2002 [1]	Vanuatu	9	100%		
	2008 [1]	Tanzania	46	100%		
[100]	2004-2011 [1]	Tanzania	390	100%		
[101]	1993 [1]	sub-Saharan Africa	13	85%	15%	
[102]	2008-2011 [1]		28	91%	9%	
[103]	2007 [1]	Kenya	253	100%		
[104]	2005-2008 [1]		78	100%		
[105]	2004 [1]		58	98%	2%	
[106]	2012 [1]	Kenya	1	100%		
		Burkina Faso	1	100%		
		Sudan	1	100%		
		Nigeria	2	100%		
		Ghana	2	100%		
[45]	2003-2010 [1]	Ghana	446	92%	8%	
[107]	2006-2007 [1]	Nigeria	16	100%		

[108]	2004 [1]	São Tomé and Príncipe	8	100%		
[109]			8	100%		
[110]	2005-2008 [1]	Benin	160	100%		
[111]	2005-2009 [1]	Cameroon	215	100%		
[112]	2006-2007 [1]	Madagascar	290	100%		
[113]	2006-2008 [1]		583	100%		
[29]	2006 [2]	Uganda	2		100%	
[93]	2007-2008 [1]		9	100%		
[114]	2010-2011 [1]		57	100%		
[115]	2007 [1]	Malawi	160	100%		
[116]	2009-2010 [1]	Senegal	174	99%	1%	
[117]	2011 [2]	Sudan	55	96%	4%	
[118]	2000-2011 [1]	KwaZulu-Natal, Limpopo, Mpumalanga	2010	100%		
[119]	2012 [1]	KwaZulu-Natal	16	100%		
[120]	2011 [1]	Ethiopia	195	100%		
[121]	2013 [1]		133	45%	55%	
[122]	2013 [1]		177	100%		
		**Asia**				
[123]	2005-2007 [1]	Pakistan	232	99%	1%	
[86]	2011 [1]		171	100%		
[87]	1992 [1]	Cambodia	10	100%		
[124]	2002-2004 [1]		224	81%	16%	3%
[125]	2004-2006 [1]		93	67%	33%	
[126]	2009-2010 [1]		182	65%	33%	2%
[127]	2011-2013 [1]		419	19%	81%	
[128]	2008-2013 [1]		644	67%	33%	
[129]	2015 [2]		12	42%	58%	

[88]	2007-2010 [1]	Afghanistan	120	100%		
[89]	2007-2010 [1]	Iran	200	100%		
[90]	2001-2002 [1]		36	100%		
[66]	1992 [1]	Myannmar, Bangladesh, Vietnam	26	88%	12%	
[66]	1992 [1]	Thailand	39	88%	12%	
[90]	2002-2008 [1]		49	53%	47%	
[130]	1990 [1]		11	9%	91%	
[27]	1990-2002 [1]		187	46%	53%	1%
[131]	1990-2002 [1]		132	30%	70%	
[132]	1993-1994 [1]		5	80%	20%	
[133]	1995-1997 [1]		54	69%	27%	4%
[134]	1997 [2]		10	50%	50%	
[135]	2000-2003 [1]		326	66%	34%	
[136]	2002-2008 [1]		46	48%	35%	17%
[137]	2006-2008 [1]		78	77%	23%	
[138]	2009 [1]		96	74%	26%	
[139]	2009 [1]		117	72%	28%	
[140]	2009 [1]		1.116	88%	12%	
[141]	2008-2009 [1]		91	40%	60%	
[142]	2007-2008 [1]	Thailand-Myanmar border	26	54%	35%	1%
[143]	2001-2002 [1]		51	90%	10%	
[144]	2010 [1]		44	64%	22%	14%
[145]	2007-2009 [1]		63	48%	24%	21%
[146]	2006-2009 [1]		123	49%	46%	5%
[147]	2002-2008 [1]		46	48%	52%	
[148]	2009-2010 [1]		168	62%	38%	
[149]	2003-2013 [1]		791	46%	54%	
[84]	2003-2005 [1]	Thailand-Myanmar border	68		100%	
		Thailand-Cambodia border	21	100%		

[85]	2003-2008 [1]	Thailand-Myanmar border	37		100%
		Thailand-Cambodia border	48	100%	
[150]	2007 [1]	Indonesia	232	86%	14%
[151]	2011-2014 [1]	Malaysia	38	100%	
[152]	2009-2010 [1]	India	75	99%	1%
[153]	2012 [1]		30	100%	
		**Oceania**			
[154]	2003-2005 [1]	Papua New Guinea	405	100%	
		**CNV in *pfgch1***			
		**Africa**			
[93]	2007-2008 [1]	Uganda	9	100%	
		**Asia**			
[28]	2000-2003 [1]	Thailand	140	28%	72%
	2002-2003 [1]	Laos	122	98%	2%

[1] Year of sample collection

[2] Date of publication of the article

[3] Number of isolates

Additional References

98. Lekweiry KM, Basco LK, Salem MS, Hafid JE, Marin-Jauffre A, Weddih AO, et al. Malaria prevalence and morbidity among children reporting at health facilities in Nouakchott, Mauritania. Trans R Soc Trop Med Hyg. 2011; 105:727-33.

99. Rijken MJ, Boel ME, Russell B, Imwong M, Leimanis ML, Phyo AP, et al. Chloroquine resistant *vivax* malaria in a pregnant woman on the western border of Thailand. Malar J. 2011; 10:113.

100. Malmberg M, Ngasala B, Ferreira PE, Larsson E, Jovel I, Hjalmarsson A, et al. Temporal trends of molecular markers associated with artemether-lumefantrine tolerance/resistance in Bagamoyo district, Tanzania. Malar J. 2013; 12:103.

101. Basco LK, Le Bras J, Rhoades Z, Wilson CM. Analysis of *pfmdr*1 and drug susceptibility in fresh isolates of *Plasmodium falciparum* from subsaharan Africa. Mol Biochem Parasitol. 1995; 74:157-66.

102. Pillai DR, Lau R, Khairnar K, Lepore R, Via A, Staines HM, et al. Artemether resistance in vitro is linked to mutations in *PfATP6* that also interact with mutations in *PfMDR1* in travellers returning with *Plasmodium falciparum* infections. Malar J. 2012; 11:131.

103. Shah M, Omosun Y, Lal A, Odero C, Gatei W, Otieno K, et al. Assessment of molecular markers for anti-malarial drug resistance after the introduction and scale-up of malaria control interventions in western Kenya. Malar J. 2015; 14:75.

104. Mwai L, Kiara SM, Abdirahman A, Pole L, Rippert A, Diriye A, et al. *In vitro* activities of piperaquine, lumefantrine, and dihydroartemisinin in Kenyan *Plasmodium falciparum* isolates and polymorphisms in *pfcrt* and *pfmdr1*. Antimicrob Agents Chemother. 2009; 53:5069-73.

105. Holmgren G, Bjorkman A, Gil JP: Amodiaquine resistance is not related to rare findings of *pfmdr1* gene amplifications in Kenya. Trop Med Int Health. 2006; 11:1808-12.

106. van Schalkwyk DA, Burrow R, Henriques G, Gadalla NB, Beshir KB, Hasford C, et al. Culture-adapted *Plasmodium falciparum* isolates from UK travellers: *in vitro* drug sensitivity, clonality and drug resistance markers. Malar J. 2013; 12:320.

107. Happi CT, Gbotosho GO, Folarin OA, Sowunmi A, Hudson T, O'Neil M, et al. Selection of *Plasmodium falciparum* multidrug resistance gene 1 alleles in asexual stages and gametocytes by artemether-lumefantrine in Nigerian children with uncomplicated *falciparum* malaria. Antimicrob Agents Chemother. 2009; 53:888-95.

108. Ferreira ID, Lopes D, Martinelli A, Ferreira C, do Rosario VE, Cravo P. *In vitro* assessment of artesunate, artemether and amodiaquine susceptibility and molecular analysis of putative

resistance-associated mutations of *Plasmodium falciparum* from Sao Tome and Principe. Trop Med Int Health. 2007; 12:353-62.

109. Ferreira ID, Rosario VE, Cravo PV. Real-time quantitative PCR with SYBR Green I detection for estimating copy numbers of nine drug resistance candidate genes in *Plasmodium falciparum*. Malar J. 2006; 5:1.

110. Bertin G, Briand V, Bonaventure D, Carrieu A, Massougbodji A, Cot M, et al. Molecular markers of resistance to sulphadoxine-pyrimethamine during intermittent preventive treatment of pregnant women in Benin. Malar J. 2011; 10:196.

111. Menard S, Morlais I, Tahar R, Sayang C, Mayengue PI, Iriart X, et al. Molecular monitoring of *Plasmodium falciparum* drug susceptibility at the time of the introduction of artemisinin-based combination therapy in Yaounde, Cameroon: implications for the future. Malar J. 2012; 11:113.

112. Andriantsoanirina V, Ratsimbasoa A, Bouchier C, Tichit M, Jahevitra M, Rabearimanana S, et al. Chloroquine clinical failures in *P. falciparum* malaria are associated with mutant *Pfmdr-1*, not *Pfcrt* in Madagascar. PLoS One. 2010; 5:e13281.

113. Andriantsoanirina V, Ratsimbasoa A, Bouchier C, Jahevitra M, Rabearimanana S, Radrianjafy R, et al. *Plasmodium falciparum* drug resistance in Madagascar: facing the spread of unusual *pfdhfr* and *pfmdr-1* haplotypes and the decrease of dihydroartemisinin susceptibility. Antimicrob Agents Chemother. 2009; 53:4588-97.

114. Ochong E, Tumwebaze PK, Byaruhanga O, Greenhouse B, Rosenthal PJ. Fitness consequences of *Plasmodium falciparum pfmdr1* polymorphisms inferred from e*x vivo* culture of Ugandan parasites. Antimicrob Agents Chemother. 2013; 57:4245-51.

115. Nkhoma S, Nair S, Mukaka M, Molyneux ME, Ward SA, Anderson TJ. Parasites bearing a single copy of the multi-drug resistance gene (*pfmdr-1*) with wild-type SNPs predominate amongst *Plasmodium falciparum* isolates from Malawi. Acta Trop. 2009; 111:78-81.

116. Wurtz N, Fall B, Pascual A, Diawara S, Sow K, Baret E, et al. Prevalence of molecular markers of *Plasmodium falciparum* drug resistance in Dakar, Senegal. Malar J. 2012; 11:197.

117. Gadalla NB, Adam I, Elzaki SE, Bashir S, Mukhtar I, Oguike M, et al. Increased *pfmdr1* copy number and sequence polymorphisms in *Plasmodium falciparu*m isolates from Sudanese malaria patients treated with artemether-lumefantrine. Antimicrob Agents Chemother. 2011; 55:5408-11.

118. Maharaj R, Morris N, Seocharan I, Kruger P, Moonasar D, Mabuza A, et al. The feasibility of malaria elimination in South Africa. Malar J. 2012; 11:423.

119. Vaughan-Williams CH, Raman J, Raswiswi E, Immelman E, Reichel H, Gate K, et al. Assessment of the therapeutic efficacy of artemether-lumefantrine in the treatment of uncomplicated *Plasmodium falciparum* malaria in northern KwaZulu-Natal: an observational cohort study. Malar J. 2012; 11:434.

120. Mekonnen SK, Aseffa A, Berhe N, Teklehaymanot T, Clouse RM, Gebru T, et al. Return of chloroquine-sensitive *Plasmodium falciparum* parasites and emergence of chloroquine-resistant *Plasmodium vivax* in Ethiopia. Malar J. 2014; 13:244.

121. Tajebe A, Aemero M, Francis K, Magoma G. Identification of chloroquine resistance *Pfcrt*-K76T and determination of *Pfmdr1*-N86Y copy number by SYBR Green I qPCR. Asian Pac J Trop Biomed. 2015; 5:208–20.

122. Heuchert A, Abduselam N, Zeynudin A, Eshetu T, Loscher T, Wieser A, et al. Molecular markers of anti-malarial drug resistance in southwest Ethiopia over time: regional surveillance from 2006 to 2013. Malar J. 2015; 14:208.

123. Ghanchi NK, Ursing J, Beg MA, Veiga MI, Jafri S, Martensson A. Prevalence of resistance associated polymorphisms in *Plasmodium falciparum* field isolates from southern Pakistan. Malar J. 2011; 10:18.

124. Lim P, Alker AP, Khim N, Shah NK, Incardona S, Doung S, et al. *Pfmdr1* copy number and arteminisin derivatives combination therapy failure in *falciparum* malaria in Cambodia. Malar J. 2009; 8:11.

125. Vinayak S, Alam MT, Sem R, Shah NK, Susanti AI, Lim P, et al. Multiple genetic backgrounds of the amplified *Plasmodium falciparum* multidrug resistance (*pfmdr1*) gene and selective sweep of 184F mutation in Cambodia. J Infect Dis. 2010; 201:1551-60.

126. Lanteri CA, Chaorattanakawee S, Lon C, Saunders DL, Rutvisuttinunt W, Yingyuen K, et al. *Ex vivo* activity of endoperoxide antimalarials, including artemisone and arterolane, against multidrug-resistant *Plasmodium falciparum* isolates from Cambodia. Antimicrob Agents Chemother. 2014; 58:5831-40.

127. Leang R, Taylor WR, Bouth DM, Song L, Tarning J, Char MC, et al. Evidence of *Plasmodium falciparum* Malaria Multidrug Resistance to Artemisinin and Piperaquine in Western Cambodia: Dihydroartemisinin-Piperaquine Open-Label Multicenter Clinical Assessment. Antimicrob Agents Chemother. 2015; 59:4719-26.

128. Chaorattanakawee S, Saunders DL, Sea D, Chanarat N, Yingyuen K, Sundrakes S, et al. *Ex Vivo* Drug Susceptibility Testing and Molecular Profiling of Clinical *Plasmodium falciparum* Isolates from Cambodia from 2008 to 2013 Suggest Emerging Piperaquine Resistance. Antimicrob Agents Chemother. 2015; 59:4631-43.

129. Chaorattanakawee S, Lanteri CA, Sundrakes S, Yingyuen K, Gosi P, Chanarat N, et al. Attenuation of *Plasmodium falciparum in vitro* drug resistance phenotype following culture adaptation compared to fresh clinical isolates in Cambodia. Malar J. 2015; 14:486.

130. Wilson CM, Volkman SK, Thaithong S, Martin RK, Kyle DE, Milhous WK, et al. Amplification of *pfmdr 1* associated with mefloquine and halofantrine resistance in *Plasmodium falciparum* from Thailand. Mol Biochem Parasitol. 1993; 57:151-60.

131. Uhlemann AC, McGready R, Ashley EA, Brockman A, Singhasivanon P, Krishna S, et al. Intrahost selection of *Plasmodium falciparum pfmdr1* alleles after antimalarial treatment on the northwestern border of Thailand. J Infect Dis. 2007; 195:134-41.

132. Mungthin M, Bray PG, Ward SA. Phenotypic and genotypic characteristics of recently adapted isolates of *Plasmodium falciparu*m from Thailand. Am J Trop Med Hyg. 1999; 60:469-74.

133. Price RN, Cassar C, Brockman A, Duraisingh M, van Vugt M, White NJ, et al. The *pfmdr*1 gene is associated with a multidrug-resistant phenotype in *Plasmodium falciparum* from the western border of Thailand. Antimicrob Agents Chemother. 1999; 43:2943-49.

134. Price R, Robinson G, Brockman A, Cowman A, Krishna S. Assessment of *pfmdr* 1 gene copy number by tandem competitive polymerase chain reaction. Mol Biochem Parasitol. 1997; 85:161-69.

135. Nair S, Nash D, Sudimack D, Jaidee A, Barends M, Uhlemann AC, et al. Recurrent gene amplification and soft selective sweeps during evolution of multidrug resistance in malaria parasites. Mol Biol Evol. 2007; 24:562-73.

136. Veiga MI, Ferreira PE, Jornhagen L, Malmberg M, Kone A, Schmidt BA, et al. Novel polymorphisms in *Plasmodium falciparum* ABC transporter genes are associated with major ACT antimalarial drug resistance. PLoS One. 2011; 6:e20212.

137. Imwong M, Dondorp AM, Nosten F, Yi P, Mungthin M, Hanchana S, et al. Exploring the contribution of candidate genes to artemisinin resistance in *Plasmodium falciparum*. Antimicrob Agents Chemother. 2010; 54:2886-92.

138. Muhamad P, Chaijaroenkul W, Congpuong K, Na-Bangchang K. SYBR Green I and TaqMan quantitative real-time polymerase chain reaction methods for the determination of amplification of *Plasmodium falciparum* multidrug resistance-1 gene (*pfmdr1*). J Parasitol. 2011; 97:939-42.

139. Muhamad P, Phompradit P, Sornjai W, Maensathian T, Chaijaroenkul W, Rueangweerayut R, et al. Polymorphisms of molecular markers of antimalarial drug resistance and relationship with artesunate-mefloquine combination therapy in patients with uncomplicated *Plasmodium falciparum* malaria in Thailand. Am J Trop Med Hyg. 2011; 85:568-72.

140. Mungthin M, Intanakom S, Suwandittakul N, Suida P, Amsakul S, Sitthichot N, et al. Distribution of *pfmdr1* polymorphisms in *Plasmodium falciparum* isolated from Southern Thailand. Malar J. 2014; 13:117.

141. Na-Bangchang K, Muhamad P, Ruaengweerayut R, Chaijaroenkul W, Karbwang J. Identification of resistance of *Plasmodium falciparum* to artesunate-mefloquine combination in an area along the Thai-Myanmar border: integration of clinico-parasitological response, systemic drug exposure, and *in vitro* parasite sensitivity. Malar J. 2013; 12:263.

142. Chaijaroenkul W, Wisedpanichkij R, Na-Bangchang K. Monitoring of *in vitro* susceptibilities and molecular markers of resistance of *Plasmodium falciparum* isolates from Thai-Myanmar border to chloroquine, quinine, mefloquine and artesunate. Acta Trop. 2010; 113:190-94.

143. Nelson AL, Purfield A, McDaniel P, Uthaimongkol N, Buathong N, Sriwichai S, et al. *pfmdr1* genotyping and *in vivo* mefloquine resistance on the Thai-Myanmar border. Am J Trop Med Hyg. 2005; 72:586-92.

144. Phompradit P, Muhamad P, Chaijaroenkul W, Na-Bangchang K. Genetic polymorphisms of candidate markers and in vitro susceptibility of *Plasmodium falciparum* isolates from Thai-Myanmar border in relation to clinical response to artesunate-mefloquine combination. Acta Trop. 2014; 139:77-83.

145. Phompradit P, Wisedpanichkij R, Muhamad P, Chaijaroenkul W, Na-Bangchang K. Molecular analysis of *pfatp6* and *pfmdr1* polymorphisms and their association with *in vitro* sensitivity in *Plasmodium falciparum* isolates from the Thai-Myanmar border. Acta Trop. 2011; 120:130-35.

146. Phompradit P, Muhamad P, Wisedpanichkij R, Chaijaroenkul W, Na-Bangchang K. Four years' monitoring of *in vitro* sensitivity and candidate molecular markers of resistance of *Plasmodium falciparum* to artesunate-mefloquine combination in the Thai-Myanmar border. Malar J. 2014; 13:23.

147. Veiga MI, Ferreira PE, Malmberg M, Jornhagen L, Bjorkman A, Nosten F, et al. *pfmdr1* amplification is related to increased *Plasmodium falciparum in vitro* sensitivity to the bisquinoline piperaquine. Antimicrob Agents Chemother. 2012; 56:3615-19.

148. Muhamad P, Chaijaroenkul W, Phompradit P, Rueangweerayut R, Tippawangkosol P, Na-Bangchang K. Polymorphic patterns of *pfcrt* and *pfmdr1* in *Plasmodium falciparum* isolates along the Thai-Myanmar border. Asian Pac J Trop Biomed. 2013; 3:931-35.

149. Phyo AP, Ashley EA, Anderson TJ, Bozdech Z, Carrara VI, Sriprawat K, et al. Declining Efficacy of Artemisinin Combination Therapy Against *P. Falciparum* Malaria on the Thai-Myanmar Border (2003-2013): The Role of Parasite Genetic Factors. Clin Infect Dis. 2016; 63:784-91.

150. Asih PB, Rogers WO, Susanti AI, Rahmat A, Rozi IE, Kusumaningtyas MA, et al. Seasonal distribution of anti-malarial drug resistance alleles on the island of Sumba, Indonesia. Malar J. 2009; 8:222.

151. Norahmad NA, Mohd Abd Razak MR, Abdullah NR, Sastu UR, Imwong M, Muniandy PK, et al. Prevalence of *Plasmodium falciparum* Molecular Markers of Antimalarial Drug Resistance in a Residual Malaria Focus Area in Sabah, Malaysia. PLoS One. 2016; 11:e0165515.

152. Pathak A, Martensson A, Gawariker S, Mandliya J, Sharma A, Diwan V, et al. Characterization of drug resistance associated genetic polymorphisms among *Plasmodium falciparum* field isolates in Ujjain, Madhya Pradesh, India. Malar J. 2014; 13:182.

153. Gupta R, Mishra N, Kumar A, Rana R, Srivastava B, Tyagi PK, et al. Monitoring artemisinin resistance in *Plasmodium falciparum*: comparison of parasite clearance time by microscopy and real-time PCR and evaluation of mutations in *Pfatpase6* gene in Odisha state of India. Parasitol Res. 2015; 114:3487-96.

154. Hodel EM, Marfurt J, Muller D, Rippert A, Borrmann S, Muller I, et al. Lack of multiple copies of *pfmdr1* gene in Papua New Guinea. Trans R Soc Trop Med Hyg. 2008; 102:1151-53.
